# Exosomes derived from 3D-cultured MSCs improve therapeutic effects in periodontitis and experimental colitis and restore the Th17 cell/Treg balance in inflamed periodontium

**DOI:** 10.1038/s41368-021-00150-4

**Published:** 2021-12-14

**Authors:** Yong Zhang, Jiayao Chen, Haijun Fu, Shuhong Kuang, Feng He, Min Zhang, Zongshan Shen, Wei Qin, Zhengmei Lin, Shuheng Huang

**Affiliations:** 1 Hospital of Stomatology, Guangdong Provincial Key Laboratory of Stomatology, Guanghua School of Stomatology, Sun Yat-sen University, Guangzhou, Guangdong China; 2 Guangzhou Digestive Disease Center, Guangzhou First People’s Hospital, School of Medicine, South China University of Technology, Guangzhou, Guangdong China; 3 The First Affiliated Hospital, Sun Yat-sen University, Guangzhou, Guangdong China; 4 Guangdong Provincial Key Laboratory of Orthopaedics and Traumatology, The First Affiliated Hospital of Sun Yat-sen University, Guangzhou, Guangdong China

**Keywords:** Mesenchymal stem cells, Stem-cell biotechnology

## Abstract

Although mesenchymal stem cell-derived exosomes (MSC-exos) have been shown to have therapeutic effects in experimental periodontitis, their drawbacks, such as low yield and limited efficacy, have hampered their clinical application. These drawbacks can be largely reduced by replacing the traditional 2D culture system with a 3D system. However, the potential function of MSC-exos produced by 3D culture (3D-exos) in periodontitis remains elusive. This study showed that compared with MSC-exos generated via 2D culture (2D-exos), 3D-exos showed enhanced anti-inflammatory effects in a ligature-induced model of periodontitis by restoring the reactive T helper 17 (Th17) cell/Treg balance in inflamed periodontal tissues. Mechanistically, 3D-exos exhibited greater enrichment of miR-1246, which can suppress the expression of Nfat5, a key factor that mediates Th17 cell polarization in a sequence-dependent manner. Furthermore, we found that recovery of the Th17 cell/Treg balance in the inflamed periodontium by the local injection of 3D-exos attenuated experimental colitis. Our study not only showed that by restoring the Th17 cell/Treg balance through the miR-1246/Nfat5 axis, the 3D culture system improved the function of MSC-exos in the treatment of periodontitis, but also it provided a basis for treating inflammatory bowel disease (IBD) by restoring immune responses in the inflamed periodontium.

## Introduction

Periodontitis is polymicrobe-induced inflammatory condition that damages the tooth-supporting tissues, including the cementum, alveolar bone, gingiva and periodontal ligament.^1^ Over 80% of people worldwide have periodontitis, which can cause tooth loosening and even tooth loss if left untreated.^2^ Additionally, periodontitis has been reported to increase the prevalence of many systemic diseases, including cardiovascular diseases, diabetes, and Alzheimer’s disease (AD).^3–5^ Recently, treatments for periodontitis have been focused on the removal of biofilm by scaling, root planning or sometimes the combination of these therapies with the administration of antibiotics.^6^ However, these treatments are often unable to remove completely periodontal pathogens, which continue to stimulate the host immune response and gradually make it unmanageable.^7–9^ In turn the dysregulated host immune-inflammatory response in the periodontium results in the further proliferation of oral pathobionts.^10^ In short, the addition of host-modulation therapy to cause-related therapy is an improved treatment strategy for periodontitis.

As mesenchymal stem cells (MSCs) can regulate host immune-inflammatory responses, an ability attributed mainly to their paracrine secretion,^11^ mesenchymal stem cell-derived exosomes (MSC-exos) are believed to held great potential in curing multiple inflammatory diseases, such as graft-versus-host disease and diabetes.^12,13^ Exosomes are extracellular vesicles secreted by cells that mediate cell-to-cell communication by the transfer of RNAs and proteins.^14,15^ Liu^16^ et al. reported that exosomes derived from bone marrow mesenchymal stem cells (BMSCs) had a therapeutic effect in rats with periodontitis. In fact, multiple researchers have found that exosomes released from MSCs from different sources can differentially alleviate periodontitis and promote periodontal regeneration.^17^ Among all sources of MSCs, dental pulp stem cells (DPSCs) have attracted particular attention because relative to BMSCs, they are readily accessible, less tumorigenic and demonstrate increased osteogenic and proliferative activity, showing greater capacity for the treatment of periodontitis.^18–20^ In this context, we have conducted a number of studies to expand the understanding of DPSCs and facilitate their clinical application.^21–24^ Recently, our group reported that dental pulp stem cell-derived exosomes (DPSC-exos) can accelerate alveolar bone healing by transferring miR-1246 to immune cells in the periodontium, which can suppress inflammatory and immune responses.^25^ Although DPSC-exos show therapeutic potential for many diseases, their low yield limits their therapeutic application.^26,27^ The yield of exosomes is affected by the culture system, isolation method and purification process employed.^26,27^ Accumulating evidence has shown that a three-dimensional (3D) culture system produces more exosomes compared with the traditional two-dimensional (2D) system.^27,28^ Moreover, compared with exosomes produced by 2D culture (2D-exos), those produced by 3D culture (3D-exos) exerted better therapeutic effects by the transfer of specific cargoes.^29^ For example, Cao^30^ et al. reported that use of a 3D culture system increased the production of exosomes by over 19-fold compared with use of a 2D culture. Yang^31^ et al. reported that compared to 2D-exos, 3D-exos exerted enhanced therapeutic effects to improve cognitive function in AD mice. Thus, we intended to investigate the therapeutic potential of DPSC-exos in 3D culture (DPSC 3D-exos) for the treatment of periodontitis.

The involvement of a “mouth-gut axis” in the pathogenesis of intestinal diseases, including inflammatory bowel disease (IBD) and intestinal tumors, has recently been widely recognized.^32–35^ The incidence of periodontitis is elevated in IBD patients,^36,37^ and periodontitis can exacerbate the severity of IBD,^38,39^ suggesting that these two inflammatory diseases are somehow both physically and pathologically connected. As a result of periodontitis, the number of periodontal pathobionts is greatly increased.^40^ These pathobionts then translocate to the intestine, where they trigger inflammation. Moreover, these periodontal pathobionts lead to the activation of reactive T helper 17 (Th17) cells in periodontal tissue;^40^ these cells are endowed with colitogenic capacity and translocate to the inflamed gut, exacerbating inflammation.^41^ Hence, it is possible that IBD could be reduced by treating periodontitis, but the extent to which colitis can be reduced in this manner is unclear.

To address the challenge of the mediocre clinical results caused by the low yield of DPSC-exos, first we attempted to improve the yield and therapeutic effect of DPSC-exos by employing a 3D culture system and explore how the 3D culture system improved the therapeutic effect of DPSC-exos on periodontitis. Second, we sought to determine whether the treatment of periodontitis can alleviate the progression of IBD. Although the connection between periodontitis and IBD has been widely reported, very few studies have provided evidence indicating the effect of periodontitis control on IBD progression. Our findings ultimately shed light on 3D-exo-based therapy for the treatment of periodontitis-enhanced colitis by suppression of the immune-inflammatory response in the inflamed periodontium.

## Results

### Characterization of 2D- and 3D-exos

**Fig. 1:**
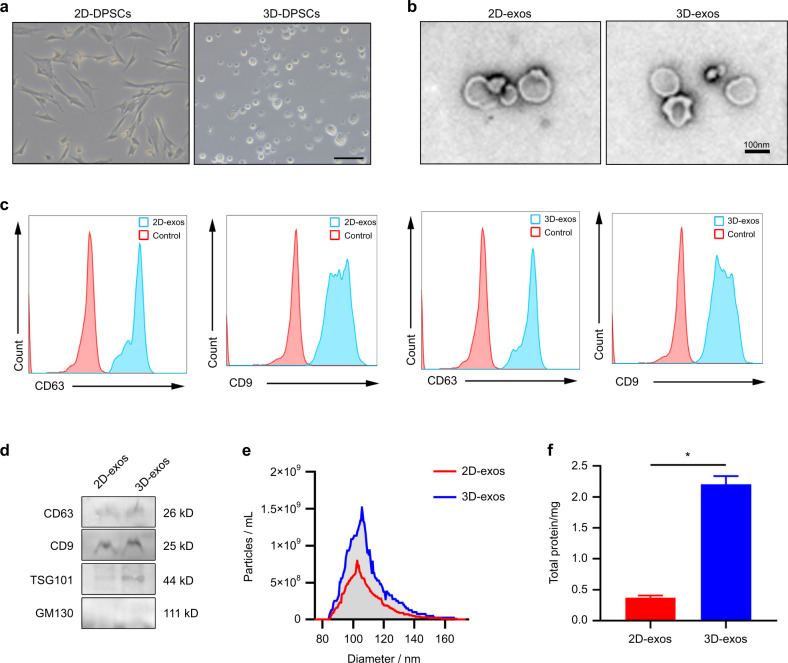
Characterization of 2D-exos and 3D-exos. **a** Brightfield images of 3D-cultured or 2D-cultured DPSCs. Scale bar = 100 μm. **b** TEM images showing the morphology of 2D-exos and 3D-exos. Scale bar = 100 nm. **c** Flow cytometric analysis showing the expression of CD63 and CD9 in 2D-exos and 3D-exos. **d** Representative western blot image showing the expression of exosome markers (CD9, CD63, and TSG101) and a cytosolic marker (GM130) in 2D-exos and 3D-exos. **e** Particle sizes and numbers of 2D-exos and 3D-exos were analyzed by using NTA. **f** BCA assays determined the yields of 2D-exos and 3D-exos

DPSCs formed multicellular spheroids in the 3D culture system, distinct from the spindle-like morphology of 2D-cultured DPSCs, which indicates that our 3D cell culture models were successfully established (Fig. 1a). Transmission electron microscopy (TEM) revealed that the 2D- and 3D-exos were nanosized vesicles with a bilayer membrane (Fig. 1b). Flow cytometric analysis demonstrated that both 2D- and 3D-exos expressed exosome markers CD63 and CD9 (Fig. 1c). Western blot analysis demonstrated that the exosome surface proteins CD63, CD9 and TSG101^42,43^ were expressed in the 2D- and 3D-exos, but the cytosolic marker GM130 was absent in the 2D- and 3D-exos (Fig. 1d).^44^ Nanoparticle tracking analysis (NTA) demonstrated that the diameters of the 2D- and 3D-exos ranged from 50 to 200 nm (Fig. 1e).^45^ Collectively, these results demonstrate that we successfully isolated 2D- and 3D-exos.

In order to investigate the yields of exosomes from DPSCs in a conventional 2D culture system and a 3D culture system, we collected supernatants from the 2D and 3D culture systems. Supernatants (at a total volume of 50 mL) were collected from 1 × 10^7^ DPSCs in 2D flasks and the 3D culture system. We utilized the BCA assay and NTA to determine the yields of 2D- and 3D-exos. The NTA results demonstrated that the number of 3D-exos from a 2-day culture of 1 × 10^7^ DPSCs was 6.36 × 10^1^per mL, while the number of 2D-exos was 3.31 × 10^10^per mL (Fig. 1e). Moreover, the BCA assay showed that the amount of 2D-exos was 0.36 mg, while the amount of 3D-exos was increased to 2.23 mg (Fig. 1f). Taken together, these results lend support to the idea that the yields of the DPSC-exos from the 3D culture system are higher than those from the 2D culture system.

### The 3D-exos exerted enhanced effects in ameliorating periodontitis and colitis

**Fig. 2:**
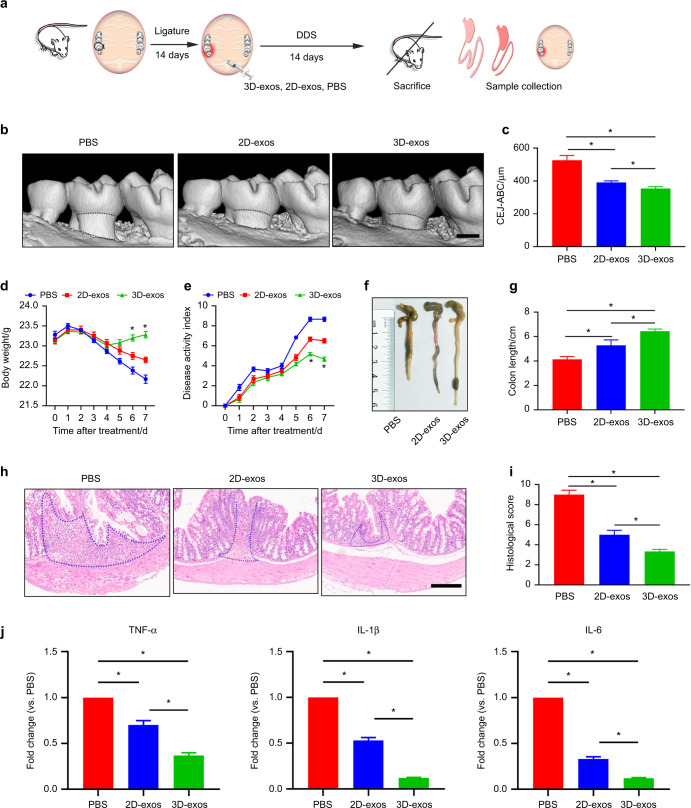
3D-exos exerted enhanced effects in ameliorating periodontitis and commensal pathobiont-driven colitis. **a** A flow diagram showing the time of ligature-induced periodontitis and DSS-induced colitis induction, periodontal injection and specimen collection. **b** 3D reconstructions of maxillae of the PBS-, 2D-exo- and 3D-exo-treated groups (*n* = 6 per group) were generated by micro-CT. The vertical line extends from the CEJ to the ABC. The CEJ-ABC distance was measured at six predetermined sites on both the buccal and palatal sides. Scale bar = 500 μm. **c** Statistical analysis of the CEJ-ABC distance in each group (*n* = 6) as determined by micro-CT. The error bars represent the SEM. *P < 0.05. **d**, **e** Analysis of body weights and disease activity index (DAI) in each group (*n* = 6). **f** Representative colon pictures of the mice in each group. **g** Statistical analysis of the colon length in each group (*n* = 6 per group). The error bars represent the SEM. *P < 0.05. **h** Histopathological changes in the colon were analyzed by haematoxylin and eosin (HE) staining. Scale bar = 200 μm. **i** Statistical analysis of histological scores in each group (*n* = 6). **j** RT-qPCR analysis of the expression levels of the *TNF-α, IL-1β* and *IL-6* genes in the colon in each group (*n* = 6). The error bars represent the SEM. *P < 0.05

To investigate the degree to which IBD can be reduced by suppressing periodontitis in this study, a ligature-induced periodontitis mouse model (P mice) and a dextran sulfate sodium (DSS)-induced colitis mouse model were used (Fig. 2a).^46^ First, we compared the therapeutic effects of 2D- and 3D-exos in the periodontitis model of DSS-P mice. The gingivae of DSS-P mice were collected after the routine injection of PBS, 2D-exos or 3D-exos into the palatal gingiva near the maxillary left second molar for 14 days (Fig. 2a). Since alveolar bone loss indicates the severity of periodontal inflammation and is a key clinical feature of periodontitis,^47^ we investigated whether the 2D-exos and 3D-exos had reduced alveolar bone resorption in the DSS-P mice. The result showed that the 3D-exo-treated group possessed the lowest alveolar bone loss, measured by micro-computed tomography (micro-CT) (3D-exos vs. PBS fold-change: 0.67 *P* = 2.24 × 10^−4^; 2D-exos vs. PBS fold-change: 0.74 *P* = 1.13 × 10^−3^; 3D-exos vs. 2D-exos fold-change: 0.91 *P* = 3.56 × 10^−2^) (Fig. 2b, c). In addition, H&E staining revealed that 3D-exo-treated group had greater volume of alveolar bone and fewer inflammatory cells in comparison with the PBS- or 2D-exo-treated groups (Fig. S1A-B). In the progression of periodontitis, TRAP^+^ osteoclast cells were involved in alveolar bone resorption.^48^ TRAP staining showed that in comparison with the PBS- or 2D-exo-treated groups, the 3D-exo-treated group had fewer osteoclasts in the periodontium (Fig. S1C-D). These results reveal that 3D-exos exert enhanced effects in ameliorating periodontitis.

Periodontal inflammation has been demonstrated to aggravate IBD.^38^ We further evaluated whether 2D- and 3D-exos could ameliorate experimental colitis by suppressing periodontitis in DSS-P mice. Significantly, the DSS-P mice gained lower disease activity index (DAI) scores and experienced less body weight loss in the 3D-exo-treated group than in the 2D-exo- or PBS-treated group (Fig. 2d, e).^49^ Shortening of the colon indicates the aggravation of intestinal inflammation.^50^ 3D-exo-treated DSS-P mice showed an increased colon length compared with 2D-exo- or PBS-treated DSS-P mice (3D-exos vs. PBS fold-change: 1.56 *P* = 9.37 × 10^−6^; 2D-exos vs. PBS fold-change: 1.28 *P* = 4.54 × 10^−2^; 3D-exos vs. 2D-exos fold-change: 1.22 *P* = 3.47 × 10^−2^) (Fig. 2f, g). Likewise, H&E staining analysis demonstrated that the 3D-exo-treated groups displayed the infiltration of fewer immune cells into the colon lamina propria, a lower degree of inflammation and a lower histological score than the 2D-exo- or PBS-treated group (Fig. 2h, i). In addition, quantitative reverse transcription PCR (RT-qPCR) results demonstrated that 3D-exos downregulated the expression of several proinflammatory genes, including *IL-1β, IL-6* and *TNF-α,*^51^ in comparison with the PBS- and 2D-exo-treated groups (Fig. 2j). Taken together, these results demonstrate that 3D-exos exert enhanced effects in ameliorating periodontitis and colitis. Notably, flow cytometric analysis and fluorescence analysis of gingiva and the colon showed that 24 h after the first injection of exosomes, abundant exosomes could be detected in the maxillary gingiva of mice but were barely detectable in intestinal tissue, consistent with the results reported by other researchers (Fig. S2).^52^ Our findings suggest that the therapeutic effect is not caused by the direct actions of the exosomes themselves on colitis but instead is achieved by exosome-mediated amelioration of periodontitis.

### 3D-exos showed enhanced anti-inflammatory efficacy in DSS-P mice

**Fig. 3:**
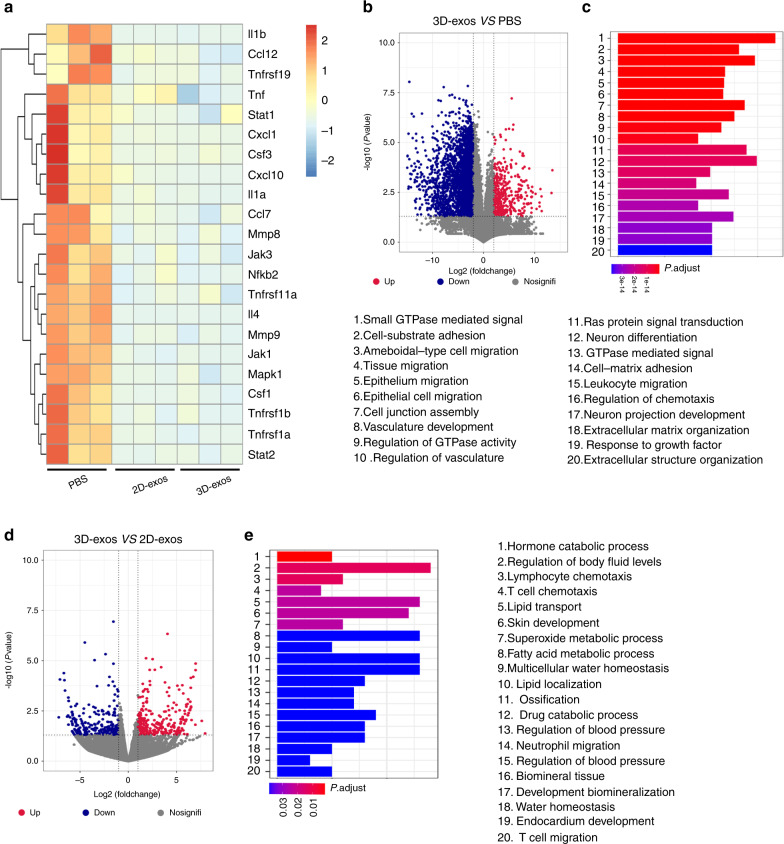
3D-exos exerted an enhanced anti-inflammatory effect in DSS-P mice. **a** Heatmap of DEGs in the gingiva of the 2D-exo- and 3D-exo-treated groups vs. the gingiva of the PBS-treated group (*n* = 3 per group). **b** Volcano plots show DEGs between the PBS- and 3D-exo-treated groups. **c** Gene Ontology (GO) functional analysis of the top 200 downregulated genes in the gingiva of the 3D-exo-treated group compared with the PBS-treated group. **d** Volcano plots show DEGs between the 2D-exo- and 3D-exo-treated groups. **e** GO functional analysis of the top 200 downregulated genes in the gingiva of the 3D-exo-treated group compared with the 2D-exo-treated group

To further investigate the effects of 3D-exos in reducing inflammation of the periodontium, we compared differential gene expression in the gingiva of the 3D-exo-treated DSS-P mice with those of the PBS- and 2D-exo-treated DSS-P mice by RNA sequencing (RNA-seq), showing that many proinflammatory genes, such as *Il1β*, *Il1α*, *Ccl12* and *Tnf*, were downregulated in both the 2D-exo- and 3D-exo-treated groups compared with the PBS-treated group (Fig. 3a). Moreover, the expression of proinflammatory genes, such as *Il1β*, *Il1α*, *Tnf* and *Ccl12*, decreased in the 3D-exo-treated group compared with 2D-exo-treated group, indicating that the 3D-exos exerted enhanced anti-inflammatory efficacy in the periodontitis model (Fig. 3a).

Furthermore, we performed Gene Ontology (GO) survey for the top 200 differentially expressed genes (DEGs) in our RNA-seq datasets. The DEGs between gingivae of 3D-exo-treated DSS-P mice and PBS-exo-treated DSS-P mice were enriched in leukocyte migration and regulation of chemotaxis (Fig. 3b, c). In addition, the DEGs between gingivae of 3D-exo-treated DSS-P mice and 2D-exo-treated DSS-P mice were enriched in T-cell chemotaxis, neutrophil migration and T-cell migration (Fig. 3d, e). These results indicate that compared with 2D-exos, 3D-exos exerted enhanced anti-inflammatory efficacy in DSS-P mice partially due to their effects in reducing inflammatory and immune cell infiltration in the gingiva of DSS-P mice.

### 3D-exos showed an enhanced ability to reduce the ratio of Th17 cells to Tregs in both the inflamed periodontium and colon

**Fig. 4:**
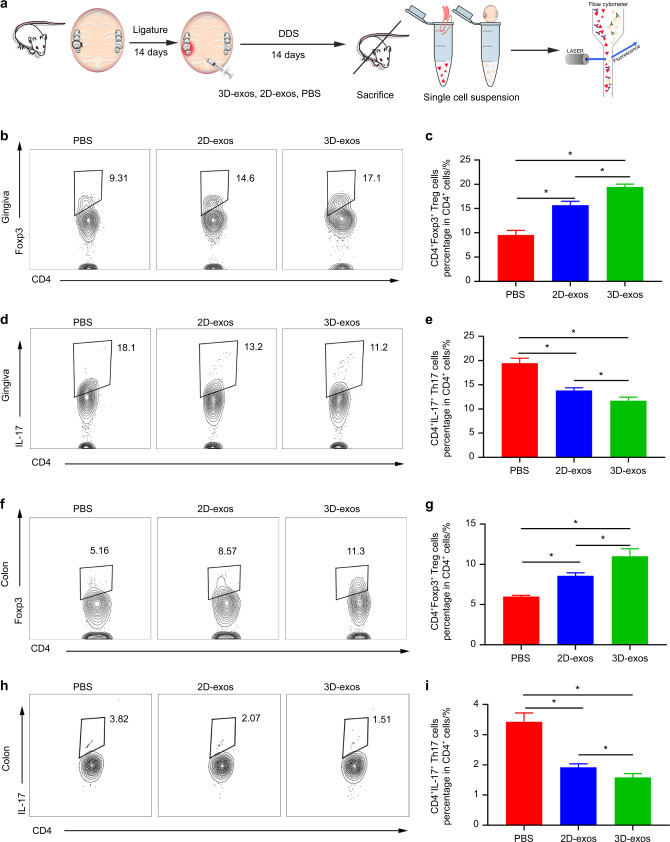
3D-exos showed an enhanced ability to reduce the ratio of Th17 cells to Treg cells in both the inflamed gingiva and the colon. **a** A flow diagram showing the time of ligature-induced periodontitis and DSS-induced colitis induction, periodontal injection, and specimen collection. **b**–**e** Flow cytometric profiles of Foxp3 and IL-17 expression in CD4^+^ cells in the inflamed gingiva of the PBS-, 2D-exo- and 3D-exo-treated groups. Numerical values denote the mean percentage of CD4^+^ cells expressing Foxp3 and IL-17 in each group (*n* = 6). The error bars represent the SEM. *P < 0.05. **f**–**i** Flow cytometric profiles of Foxp3 and IL-17 expression in CD4^+^ cells in the inflamed colon of the PBS-, 2D-exo- and 3D-exo-treated groups. Numerical values denote the mean percentage of CD4^+^ cells expressing Foxp3 and IL-17 in each group (*n* = 6). The error bars represent the SEM. *P < 0.05

The infiltration of CD4^+^ T cells has been reputed to participate in the aggravation of periodontitis and colitis.^53,54^ Tregs and Th17 cells are subpopulations of CD4^+^ T cells.^55^ Disturbance of the balance between Th17 cells and Tregs has been found to aggravate periodontitis and colitis.^56,57^ Thus, we investigated the ability of the 3D-exos to reduce the Th17 population and increase the Treg population in the inflamed periodontium (Fig. 4a). Flow cytometric data showed that there were fewer Th17 cells (3D-exos vs. PBS fold-change: 0.61 *P* = 1.1 × 10^−4^; 2D-exos vs. PBS fold-change: 0.71 *P* = 7.4 × 10^−4^; 3D-exos vs. 2D-exos fold-change: 0.85 *P* = 4.45 × 10^−2^) and more Tregs in the gingiva of the 3D-exo-treated group compared with the 2D-exo-treated and PBS-treated groups (3D-exos vs. PBS fold-change: 2.03 *P* = 4 × 10^−6^; 2D-exos vs. PBS fold-change: 1.64 *P* = 6.13 × 10^−4^; 3D-exos vs. 2D-exos fold-change: 1.24 *P* = 3.3 × 10^−3^) (Fig. 4b–e and Fig. S3).

The transfer of oral Th17 cells to the colon has been found to exacerbate colitis.^41^ Flow cytometric data showed that there were fewer Th17 cells (3D-exos vs. PBS fold-change: 0.45 *P* = 1.2 × 10^−4^; 2D-exos vs. PBS fold-change: 0.56 *P* = 6.87 × 10^−4^; 3D-exos vs. 2D-exos fold-change: 0.83 *P* = 4.42 × 10^−2^) and more Tregs in the inflamed colon of the 3D-exo-treated group compared with the 2D-exo-treated and PBS-treated groups (3D-exos vs. PBS fold-change: 1.83 *P* = 3.4 × 10^−4^; 2D-exos vs. PBS fold-change: 1.43 *P* = 7.62 × 10^−5^; 3D-exos vs. 2D-exos fold-change: 1.28 *P* = 3.54 × 10^−2^) (Fig. 4f–i and Fig. S4). Taken together, these findings show that 3D-exos showed an enhanced ability to reduce the Th17 cell population and increase the Treg population in both the inflamed periodontium and colon compared with those of PBS and 2D-exos.

### 3D-exos exhibited an intensified ability to inhibit Nfat5 in T cells by miR-1246

**Fig. 5:**
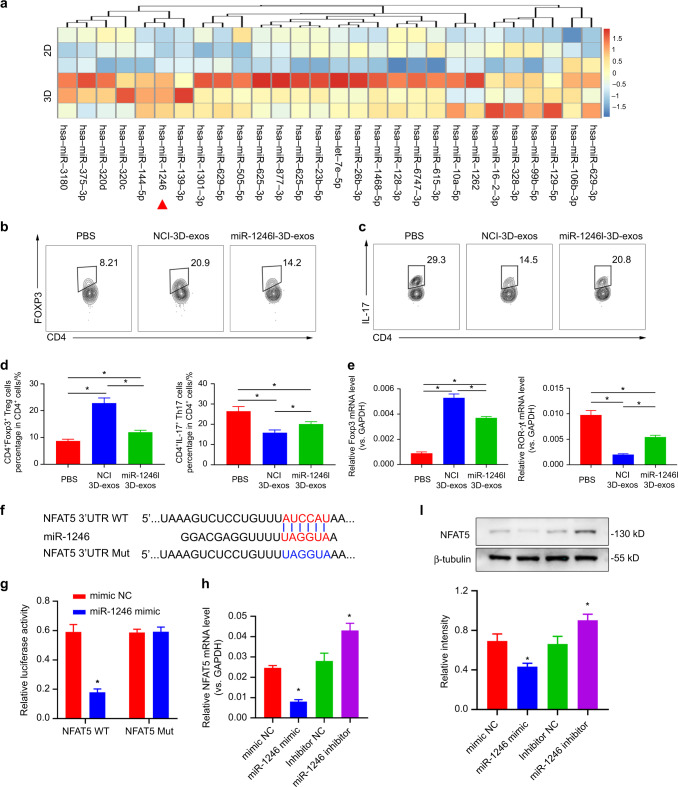
3D-exos showed an enhanced ability to inhibit Nfat5 in T cells by miR-1246. **a** Heatmap of the relative proportion of miRNAs in the total miRNA reads of the 3D-exo group vs. the 2D-exo group (*n* = 3 per group). **b**–**d** CD4^+^ naive T cells were stimulated with the indicated cytokines to induce Treg cells and Th17 cells and then treated with PBS, NCI-3D-exos or miR1246I-3D-exos in vitro. Flow cytometric profiles of Foxp3 and IL-17 expression in the above CD4^+^ T cells. Numerical values denote the mean percentage of CD4^+^ cells expressing Foxp3 and IL-17 in each group (*n* = 6). The error bars represent the SEM. *P < 0.05. **e** RT-qPCR analysis of the expression of the Treg-associated gene *Foxp3* and the Th17 cell-associated gene *ROR-γt* in CD4^+^ T cells of the PBS-, NCI-3D-exo- and miR-1246I-exo-treated groups. **f** Binding sites in the Nfat5 3′-UTR targeted by miR-1246. **g** The wild-type binding site sequence of Nfat5 was cotransfected with miR-1246, which led to decreased luciferase activity, verifying their targeted relationship. **h** RT-qPCR analysis of the mRNA expression of *Nfat5* in CD4^+^ T cells after transfection of the miR-1246 mimic and miR-1246 inhibitor. **i** Western blot analysis of the expression of Nfat5 in CD4^+^ T cells after transfection with the miR-1246 mimic and miR-1246 inhibitor

miRNAs in exosomes derived from stem cells are vital in cell-to-cell communication.^58^ We utilized miRNA sequencing (miRNA-seq) to compare the exosomal miRNAs in 3D- and 2D-exos. Compared with 2D-exos, miRNA-seq showed twenty-eight differentially accumulated miRNAs in 3D-exos. Of note, miR-1246 was the most differentially expressed miRNA (8.58-fold) in 3D-exos (Fig. 5a). RT-qPCR analysis confirmed the levels of miR-1246 in 3D-exos and 2D-exos (Fig. S5). Furthermore, naïve CD4^+^ T cells were stimulated to induce the formation of Tregs and Th17 cells in vitro. These cells were further treated with 3D-exos with the negative control inhibitor (NCI-3D-exos) or the miR-1246 inhibitor (miR1246I-3D-exos) to investigate the roles of miR-1246 in Treg and Th17 cell differentiation. Flow cytometric data showed increased Th17 cell differentiation (NCI-3D-exo group vs. PBS fold-change: 0.6 *P* = 2.56 × 10^−3^; miR1246I-3D-exo group vs. PBS fold-change: 0.76 *P* = 3.29 × 10^−2^; miR1246I-3D-exo group vs. NCI-3D-exo group fold-change: 1.27 *P* = 0.38 × 10^−2^) and decreased Treg differentiation in miR1246I-3D-exo-treated splenic T cells compared with NCI-3D-exo-treated splenic T cells (NCI-3D-exo group vs. PBS fold-change: 2.61 *P* = 4.01 × 10^−5^; miR1246I-3D-exo group vs. PBS fold-change: 1.37 *P* = 8.1 × 10^−3^; miR1246I-3D-exo group vs. NCI-3D-exo group fold-change: 0.52 *P* = 3.71 × 10^−4^) (Fig. 5b–d and Fig. S6). Likewise, RT-qPCR data revealed a reduction in the expression of the Treg-associated gene *Foxp3*^59^ and an increased expression level of the Th17 cell-associated gene *ROR-γt*^60^ in splenic T cells treated with miR1246I-3D-exos (Fig. 5e). These results indicate that the 3D-exos reduced the Th17 population and increased the Treg population partially via miR-1246.

To investigate the mechanisms by which miR-1246 downregulated Th17 cell-associated genes, we predicted target genes of miR-1246 by using TargetScan. TargetScan predicted that miR-1246 can target Nfat5 (Fig. 5f), which is a potent inducer of Th17 cells and upregulates *Il17* and *ROR-γt* expression in T cells.^61^ Moreover, luciferase reporter data straightforwardly revealed that miR-1246 can target Nfat5. Transfection of a miR-1246 mimic reduced the luciferase activity of T cells expressing Nfat5 (Fig. 5g). To investigate the roles of miR-1246 in the expression of Nfat5 in CD4^+^ T cells, we transferred miRNA mimic/inhibitor into CD4^+^ T cells. RT-qPCR and Western blot data demonstrated that Nfat5 was downregulated in CD4^+^ T cells after the transfer of the miR-1246 mimic but upregulated after the transfer of the miR-1246 inhibitor (Fig. 5h, i). As Nfat5 activation has been reported to promote Th17 cell polarization, the enhanced ability of 3D-exos to inhibit Th17 cell polarization from CD4^+^ T cells may occur via downregulation of *Nfat5* gene expression by miR-1246.

### mir-1246 antagomir reversed the effects of 3D-exos in ameliorating periodontitis and colitis

**Fig. 6:**
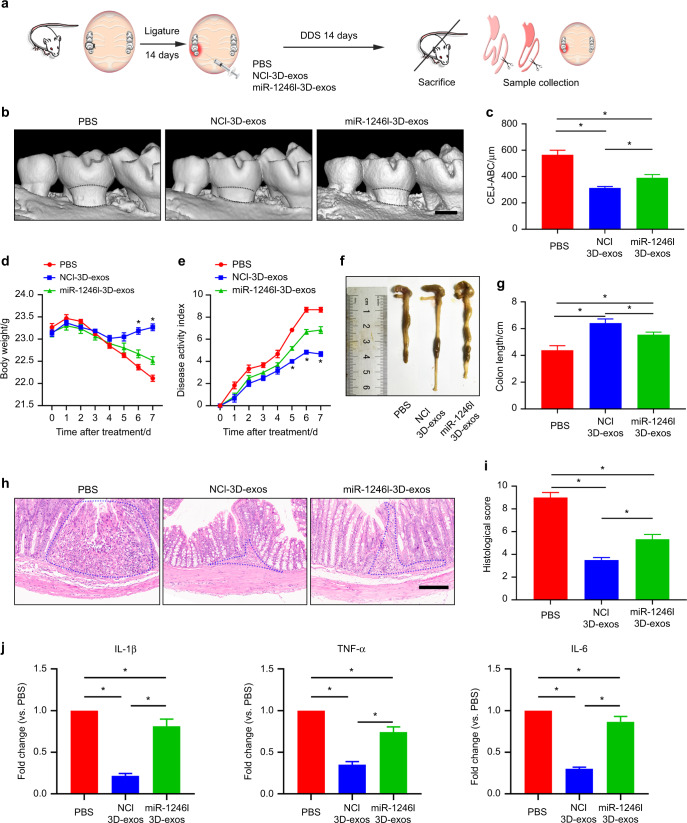
mir-1246 antagomir reversed the effects of 3D-exos in ameliorating periodontitis and commensal pathobiont-driven colitis. **a** A flow diagram showing the time of animal model induction, periodontal injection, and specimen collection. **b** 3D reconstructions of maxillae from the PBS-, NCI-3D-exo-, and miR1246I-3D-exo-treated groups (*n* = 6 per group) were generated by micro-CT. The CEJ-ABC distance was measured. Scale bar = 500 μm. **c** Statistical analysis of the CEJ-ABC distance in each group (*n* = 6 per group) as determined by micro-CT. The error bars represent the SEM. *P < 0.05. **d**, **e** Statistical analysis of body weights and DAI scores of the mice in each group (*n* = 6). **f** Representative images of colons from experimental mice in each group. **g** Statistical analysis of the colon length in each group (*n* = 6). The error bars represent the SEM. *P < 0.05. **h** Histopathological changes in colon tissues were analyzed by haematoxylin and eosin (HE) staining. Scale bar = 200 μm. **i** Statistical analysis of the histological score in each group (*n* = 6). **j** RT-qPCR was used to analyze expression levels of the *TNF-α, IL-1β*, and *IL-6* genes in each group (*n* = 6). The error bars represent the SEM. *P < 0.05

To further confirm that miR-1246 in 3D-exos contributes to restoring the Th17 cell/Treg balance in periodontitis, we treated DSS-P mice with 3D-exos with the miR-1246 antagomir (miR1246I-3D-exos) or NC antagomir (NCI-3D-exos) (Fig. 6a).^62^ Micro-CT analysis revealed more alveolar bone loss in the DSS-P mice treated with miR1246I-3D-exos than in those treated with NCI-3D-exos (NCI-3D-exo group vs. PBS fold-change: 0.55 *P* = 4.01 × 10^−5^; miR1246I-3D-exo group vs. PBS fold-change: 0.68 *P* = 2.07 × 10^−3^; miR1246I-3D-exo group vs. NCI-3D-exo group fold-change: 1.25 *P* = 1.37 × 10^−2^) (Fig. 6b, c). Furthermore, H&E staining analysis demonstrated increased infiltrating inflammatory cells and decreased alveolar bone volume in the miR1246I-3D-exo group compared with the NCI-3D-exo group (Fig. S7A-B). TRAP staining analysis showed that more osteoclasts resided in the periodontium of the miR1246I-3D-exo group than the NCI-3D-exo group (Fig. S7C-D).

A similar finding was detected in DSS-P mice with IBD. The miR1246I-3D-exo group showed more severe colitis than the NCI-3D-exo group or PBS group. Compared with DSS-P mice treated with NCI-3D-exos, DSS-P mice treated with miR1246I-3D-exos showed greater body weight loss and had higher DAI scores (Fig. 6d, e), with a shortened colon length (NCI-3D-exo group vs. PBS fold-change: 1.46 *P* = 1.27 × 10^−3^; miR1246I-3D-exo group vs. PBS fold-change: 1.26 *P* = 1.39 × 10^−2^; miR1246I-3D-exo group vs. NCI-3D-exo group fold-change: 0.86 *P* = 3.93 × 10^−2^) (Fig. 6f, g). In parallel, H&E staining analysis illustrated higher immune infiltration in the colon and higher histological scores in the colon in the miR1246I-3D-exo group than in the 3D-exo group (Fig. 6h, i). RT-qPCR analysis revealed a higher degree of inflammatory factor expression in the colon in the miR1246I-3D-exo group than in the NCI-3D-exo group (Fig. 6j).

**Fig. 7:**
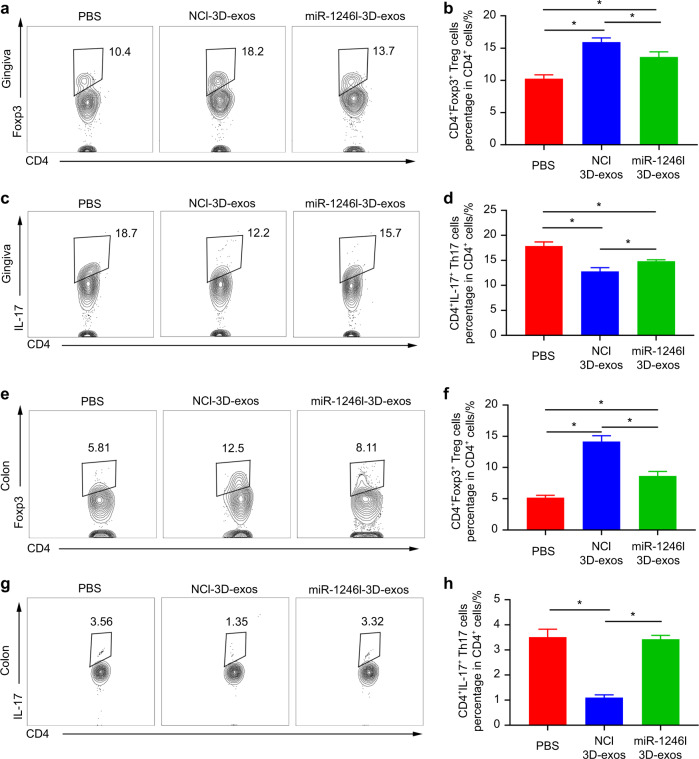
mir-1246 antagomir reversed the effects of 3D-exos, reducing the ratio of Th17 cells to Treg cells in both the inflamed gingiva and colon. **a**–**d** Flow cytometric profiles of Foxp3 and IL-17 expression in CD4^+^ cells in the inflamed gingiva of PBS-, 3D-exo– and 3D-exo+ antagomir 1246-treated groups. Numerical values denote the mean percentage of CD4^+^ cells expressing Foxp3 and IL-17 in each group (*n* = 6). The error bars represent the SEM. *P < 0.05. **e**–**h** Flow cytometric profiles of Foxp3 and IL-17 expression in CD4^+^ cells in the inflamed colon of the PBS-, NCI-3D-exo-, and miR1246I-3D-exo-treated groups. Numerical values denote the mean percentage of CD4^+^ cells expressing Foxp3 and IL-17 in each group (*n* = 6). The error bars represent the SEM. *P < 0.05

Moreover, inhibition of miR-1246 weakened the immunomodulatory function of 3D-exos. Flow cytometric analysis of periodontal cells revealed an increase in the Th17 cell population (NCI-3D-exo group vs. PBS fold-change: 0.72 *P* = 9.94 × 10^−4^; miR1246I-3D-exo group vs. PBS fold-change: 0.83 *P* = 5.51 × 10^−3^; miR1246I-3D-exo group vs. NCI-3D-exo group fold-change: 1.16 *P* = 2.89 × 10^−2^) and a decrease in the Treg population in the gingiva of the miR1246I-3D-exo group compared with the NCI-3D-exo group (NCI-3D-exo group vs. PBS fold-change: 1.55 *P* = 7.51 × 10^−5^; miR1246I-3D-exo group vs. PBS fold-change: 1.33 *P* = 7.11 × 10^−3^; miR1246I-3D-exo group vs. NCI-3D-exo group fold-change: 0.86 *P* = 4.74 × 10^−2^) (Fig. 7a–d and Fig. S3). Additionally, the Th17 population was increased (NCI-3D-exo group vs. PBS fold-change: 0.31 *P* = 2.7 × 10^−5^; miR1246I-3D-exo group vs. PBS fold-change: 0.98 *P* = 0.813; miR1246I-3D-exo group vs. NCI-3D-exo group fold-change: 3.11 *P* = 1.97 × 10^−7^), while the Treg population was reduced in the colon of the miR1246I-3D-exo group compared with the NCI-3D-exo group (NCI-3D-exo group vs. PBS fold-change: 2.74 *P* = 4.4 × 10^−6^; miR1246I-3D-exo group vs. PBS fold-change: 1.67 *P* = 1.51 × 10^−3^; miR1246I-3D-exo group vs. NCI-3D-exo group fold-change: 0.61 *P* = 8.57 × 10^−3^) (Fig. 7e–h and Fig. S4). All of these results indicate that miR-1246 mediates the immunomodulatory effect of 3D-exos, which can modulate the Th17 cell/Treg balance to ameliorate periodontitis and colitis.

## Discussion

The Th17 cell/Treg balance is crucial for the proper host defensive response to pathogens in tissues such as the periodontium and colon, where most commensal microbes reside.^63,64^ The disturbance of the Th17 cell/Treg balance has become the leading cause of various inflammatory diseases, such as periodontitis and IBD. In this study, we discovered that 3D culture endowed DPSC-exos with an enhanced ability to restore the Th17 cell/Treg balance to alleviate symptoms of periodontitis. Furthermore, we explored the mechanisms behind this strengthened anti-inflammatory effect of 3D-exos compared with 2D-exos and found that exosomal miR-1246 was increased in 3D-exos compared with 2D-exos and that this increase in miR-1246 substantially downregulated Nfat5 to modulate the functions of CD4^+^ T cells by decreasing Th17 cell differentiation. Finally, along with the alleviation of periodontitis, the severity of IBD was drastically reduced after the Th17 cell/Treg balance in the periodontium was restored via 3D-exo treatment.

MSCs exert anti-inflammatory effects via paracrine signals, and exosomes are one of the mediators of these effects.^65^ Kim^66^ reported that exosomes derived from MSCs can treat ischemic stroke by promoting a number of therapeutic effects, including the anti-inflammatory response, in ischemic brain lesions. Our preliminary study also showed that application of DPSC-exos to the gingiva can alleviate periodontitis.^25^ However, the traditional production of exosomes based on 2D adherent monolayer cell culture generates a low yield, requiring hundreds of culture flasks to yield enough exosomes for animal experiments, and the therapeutic effect of exosomes produced in this way is often inconsistent in large randomized controlled studies, strongly indicating the need to further optimize exosome production.^30^ The preparation of MSCs as spheroids has emerged as one such optimization method.^27^ A 3D spheroid culture system inhibits cells anchoring to the plastic surface of the culture flasks so that the cells can form aggregates in suspension.^67^ The alteration of cell-to-cell contacts and the modified architecture of materials around the cells may ultimately change their characteristics, such as their secretory behavior.^28,31,68^ Cao reported that MSC-exos were obtained at a higher yield in a 3D culture system than in a 2D culture system, and the 3D-cultured exosomes showed an enhanced anti-inflammatory effect that protected the kidney from cisplatin-induced injury.^30^ Our current study demonstrated that the 3D culture of DPSCs in an ultra-low-attachment tissue culture flask enabled the production of DPSCs at a higher yield than 2D-cultured DPSCs. We also found more effective alleviation of periodontitis in the 3D-exo group whose periodontium contained fewer osteoclasts and infiltrated inflammatory cells; in addition, compared with the 2D-exo group, this group exhibited decreased proinflammatory factor expression and alveolar bone loss, indicating that 3D-exos exerted a stronger anti-inflammatory effect against periodontitis and a more powerful therapeutic effect on restoring histopathological damage in periodontitis.

To investigate the mechanism underlying the enhanced anti-inflammatory property of 3D-exos, we compared the results of RNA-seq analysis of the inflamed periodontium under conditions of 2D-exo or 3D-exo treatment. GO analysis showed that most of the DEGs were related to T-cell migration. We also found that fewer CD4^+^ T cells had infiltrated the periodontium of 3D-exo-treated DSS-P mice. These results suggest that 3D-exos may modulate periodontitis by affecting T cells. Previous studies have indicated that a number of inflammatory diseases, including rheumatoid arthritis (RA) and IBD, are correlated with an imbalance in Th17 cell/Treg-related cytokines.^69,70^ An excessive Th17 cell response to pathogens induces increased proinflammatory cytokine IL-17 expression, which in turn stimulates other cells, such as epithelial cells, to produce IL-6, TNF-α, and IL-1, ultimately perpetuating inflammation.^54^ However, Th17 cell-induced inflammation can be counterbalanced by Tregs. As a suppressive subset of T cells, Tregs express TGF-β and other protective anti-inflammatory cytokines to mediate the overactivation of Th17 cells, limit injury to inflamed tissue and facilitate repair.^53^ In this context, a Th17 cell/Treg imbalance has been reported in periodontitis patients,^71^ and a number of researchers, such as Elashiry^52^ et al., have found that periodontitis can be alleviated by shifting the Th17 cell/Treg balance, indicating that the this balance contributes to maintaining periodontal homeostasis. In our study, 3D-exos suppressed Th17 cell differentiation and increased Foxp3 expression, which is considered the “master regulator” of Treg development, suggesting that our 3D-exos are a potential therapeutic strategy for abrogating the Th17 cell/Treg imbalance.

Interestingly, as periodontitis was ameliorated, IBD was also significantly alleviated after treatment with the exosomes, especially in 3D-exo-treated DSS-P mice, which exhibited decreases in proinflammatory cytokine expression, immune cell infiltration and colon shortening, accompanied by an increase in body weight. IBD is a clinically intractable challenge that emerges at a young age and persists for life.^72^ Considering the important role of Th17 cells/IL-17 in IBD onset, several drugs that either inhibit Th17 cell activation by neutralizing IL-12/IL-23 or block IL-17 cell signaling via antibodies have been developed.^73^ However, treatment resistance and the risk of immunogenicity remain major drawbacks; thus, the need to identify alternative therapeutic strategies for IBD treatment is urgent.^54^ Kitamoto^41^ reported that pathogenic Th17 cells that accumulated in the intestinal mucosa of IBD mice were derived from the oral cavity; these cells arose and multiplied during periodontitis and then transmigrated to the colon mucosa. Accordingly, our study found that along with a reduction in the Th17 cell population in inflamed periodontal tissues, these cells were also decreased in intestinal lesions of the 3D-exo-treated DSS-P mice, and the colon showed less inflammation. Our work not only supports the previously proposed idea of interplay between periodontitis and IBD but also identifies periodontitis control as an innovative therapeutic approach for IBD.

Notably, the alteration in the functions of MSC-exos acquired from the 3D culture system may be indicated by their unique cargo (miRNAs and/or proteins).^27^ Yang^31^ et al. reported that by upregulation of specific miRNAs (e.g., miR-135a) encapsulated in MSC-exos, the 3D culture system exerted enhanced therapeutic effects in ameliorating the cognitive deficits. However, the mechanism underlying this effect of the 3D culture system on the functionalities of DPSC-exos remains unknown. Given recent studies demonstrating that several miRNAs, such as miR-146a and miR-155-5p, are involved in maintaining the Th17 cell/Treg balance,^74,75^ we speculated that 3D-exos may also rely on miRNAs for enhancement of their T-cell modulatory function and applied miRNA-seq to compare the miRNA profiles of 3D-exos and 2D-exos. miR-1246 emerged as a candidate of interest in DPSC-exos because its expression was 6.2-fold higher in 3D-exos than in 2D-exos, and miR-1246 was also predicted by bioinformatics analyses to target Nfat5. Given the critical function of Nfat5 in promoting Th17 cell polarization,^61,76,77^ we hypothesized that 3D-exos restore periodontal immunological homeostasis through miR-1246, which targets Nfat5 in recipient T cells to restore the Th17 cell/Treg balance. We observed that unlike NCI-3D-exos, miR1246I-3D-exos suppressed Treg differentiation, increased the number of Th17 cells, and failed to prevent periodontitis-related alveolar bone loss and IBD in DSS-P mice, strongly indicating the indispensable role of miR-1246 in the modulatory effect of 3D-exos on T-cell differentiation and their ability to inhibit the progression of periodontitis and IBD. In addition, we found that miR-1246 mimics downregulated Nfat5 expression in CD4^+^ T cells, as expected, while the absence of miR-1246 resulted in upregulation of Nfat5. The luciferase reporter assay also confirmed that Nfat5 and miR-1246 are directly connected. Taken together, these results indicate that by targeting Nfat5, miR-1246 transferred by DPSC-exos contributes to restoring the Th17 cell/Treg balance, thereby alleviating periodontitis and IBD. However, the exact mechanism driving this increase in miR-1246 in exosomes remains incompletely understood.

Our data showed that DPSCs form multicellular spheroids in 3D culture. Due to this spheroidal structure, the surrounding cell microenvironment, including oxygen content, diffusion of nutrients and waste, and enhanced cell-to-cell cadherin binding, can be altered, and these alterations may subsequently modulate the cellular response and influence cell behaviors.^67^ Considering the importance of miR-1246 in our study, we are intensively investigating the mechanism by which the 3D culture system regulates the miR-1246 content in exosomes. Notably, hypoxia, which can increase the miR-1246 level in exosomes via selective packaging and upregulating miR-1246 expression,^78^ is also a well-recognized feature of the internal core of spheroids.^79^ Hence, the increased level of miR-1246 in 3D-exos may result from the hypoxic conditions generated by the spheroidal structure of DPSCs in the 3D culture system. A second mechanism could occur through the TP53, a well-known transcription factor of miR-1246.^80^ Recent studies have shown that as a key transcription factor of CDH1 that participates in the formation of MSC spheroids,^67,81^ the TP53 status is crucial in determining the morphology and size of the spheroidal structures.^82^ Hence, when DPSCs form aggregates under low-attachment conditions through enhanced cadherin binding induced by TP53, TP53 may simultaneously increase the expression of miR-1246, leading to its increased level in exosomes. Another suggested mechanism acts on the epigenetic level. It has been reported that histone H3 lysine 9 acetylation (H3K9ac), which generally promotes transcriptional activation, is upregulated in the promoter region of *POU5F1* in MSC spheroids.^79^ As POU5F1 is another identified transcription factor of miR-1246,^78^ the 3D culture system may increase the miR-1246 level through altering the openness of *POU5F1* chromatin and increasing its expression. Importantly, these mechanisms are merely speculative, and further studies are required to reveal the exact mechanism underlying the increase in miR-1246 in 3D-exos.

This work has some limitations that require consideration. According to other studies, the cargo contents of 2D-exos and 3D-exos are substantially different in terms of not only miRNAs but also other noncoding RNAs or proteins,^28^ and thus, further RNA-seq and proteomic analyses should be employed to fully understand the mechanism behind the strengthened therapeutic properties of 3D-exos. Another limitation relates to the pathological connection between the periodontium and colon, as intestinal colonization of oral microbes is another major pathway by which periodontitis exacerbates IBD.^83,84^ Therefore, subsequent studies to evaluate these bacteria are needed to obtain more evidence on the role of 3D-exos in periodontitis and IBD.

In conclusion, our work indicates that 3D culture conditions can improve the production of DPSC-exos and enhance their anti-inflammatory properties against periodontitis in comparison with the 2D culture conditions. The 3D-exos contained more miR-1246, which can mediate CD4^+^ T-cell differentiation by decreasing Nfat5, thereby relieving Th17 cell/Treg imbalance and alleviating periodontitis. In addition, we are the first to provide strong evidence that the restoration of this Th17 cell/Treg imbalance by 3D-exos in severe periodontal disease can reduce the severity of IBD. These results indicate the importance of periodontitis management during the optimization of therapeutic strategies for IBD treatment. Our study has revealed that the use of exosomes derived from 3D-cultured MSCs may be a promising therapeutic approach for immune balance in both periodontitis and IBD.

## Materials and methods

### DPSC culture

The use of teeth and the study protocols were approved by the Institutional Ethics Committee Board of the Guanghua School of Stomatology, Sun Yat-sen University (KQEC-2019-06). Informed consent was obtained from the donor patient family. We obtained exfoliated third molars from healthy donors 18-24 years in age who provided informed consent. DPSCs were isolated as previously described. Briefly, the pulp in the chamber and canals was gently removed using various instruments and cut into small fragments. The dental pulp tissue was added to a solution of collagenase type I (4 mg·mL^−1^; Sigma-Aldrich, MO, USA) and dispase (4 mg·mL^−1^; Sigma-Aldrich). The solution was placed at 37 °C for 30 min, with the tube inverted at 10-min intervals. The single-cell suspension was cultured in low-glucose Dulbecco’s modified Eagle’s medium (Gibco, Grand Island, NY, USA) containing 20% fetal bovine serum (Gibco) and 1% penicillin/streptomycin (Sigma-Aldrich) in a 60 mm culture flask (Corning, Cambridge, MA, USA) or 60 mm ultra-low-attachment culture dish (Corning) at 37 °C in a 5% CO_2_ humidified atmosphere.

### Isolation of DPSC-exos

To prepare exosome-depleted fetal bovine serum (FBS), FBS was ultracentrifuged at 4 °C at 120 000 × *g* for 18 h. The supernatant was filtered using a 0.22-μm syringe filter and stored at 4 °C. For 2D-exo collection, as described in a previously published protocol,^25^ cells were cultivated in Dulbecco’s modified Eagle medium containing 10% exosome-depleted FBS for 2 days. The same amount of MSCs used for 2D culture was used for inoculation in an ultra-low-attachment culture dish (Corning) for 3D culture with the same complete medium used for 2D culture for 2 days. After the culture supernatants were collected, exosomes from the 2D and 3D cultures were isolated through multistep centrifugation as previously described.^25^ To erase debris and dead cells, we first centrifuged the supernatant at 300 × *g* for 10 min, 2 000 × *g* for 20 min, and 10 000 × *g* for 30 min. Then, we ultracentrifuged the supernatant at 100 000 × *g* for 90 min and washed the pellet with PBS before centrifugation at 100 000 × *g* for 90 min (Optima-90 K, Beckman Coulter). At last, we resuspended the pellets in PBS.

Exosomes were characterized by TEM, NTA, and western blot analysis of exosome markers. The morphology and ultrastructure of the exosomes were analyzed using TEM (JEOL, Tokyo, Japan). The yields of exosomes from 1 × 10^7^ MSCs cultivated in the 2D and 3D culture systems were quantified by a Micro Bicinchoninic Acid Protein Assay Kit (CWBio, Beijing, China) and NTA according to the manufacturer’s recommended protocol. CD63 and TSG101 protein levels were determined using Western blot analysis. The therapeutic effects of 3D-exos and 2D-exos were examined in a mice model of periodontitis and DSS-induced colitis in vivo and in vitro.

To understand the distribution of the exosomes after injection, exosomes were stained with DiO (Invitrogen, USA), a fluorescent dye that can label the plasma membrane. At 24 h after the first injection of exosomes into the palatal gingiva near maxillary left second molar of DSS-P mice, gingivae from the maxilla and colons were collected. They were either fixed in 4% PFA and cut into frozen sections for confocal fluorescence analysis or treated with enzymatic digestion (RPMI-1640 medium containing 4 mg/mL dispase and 3 mg·mL^−1^ collagenase type I) for flow cytometric analysis. The digest solution was then filtered through a 70-μm cell strainer (Biologix Research Company, USA) to obtain a single-cell suspension. The ex vivo fluorescence intensity was analyzed by flow cytometry.

### Animals

Six- to eight-week-old male C57BL/6J mice were purchased from the National Resource Center of Model Mice (Nanjing, China). With the importance of IBD alleviation accounted for in our study, the sample size was estimated by using one-way analysis of variance F-tests with the mean difference of the DAI of the colon representing the severity of colitis among the PBS-treated group, 2D-exo-treated group and 3D-exo-treated group. We estimated at least 6 mice are required to reach a statistical power of 90% with type I error at 5%. The sample size was calculated using PASS software. All mice were maintained under specific pathogen-free conditions in an environmentally controlled clean room at the Center for Experimental Medicine. All experiments were performed with the approval of the Animal Care and Use Committee of Sun Yat-sen University (SYSU-IACUC-2019-000096). A total of 5 experimental groups are described and listed in Table S3. Each group was comprised of 6 mice.

### Ligature-induced periodontitis model

The mouse was anaesthetized with 4% isoflurane flow (RWD, Shenzhen, Guangdong, China). Then, a ligature (5-0 silk) was placed around the maxillary left second molar from day 0 to day 14, as described previously.^25^ After 14 days, PBS, 2D-exos, 3D-exos, NCI-3D-exos or miR1246I-3D-exos (50 μg per mouse) was injected into the palatal gingiva of the experimental mice over a period of 14 days (once every 7 days). The mice were sacrificed and analyzed 14 days after PBS or exosome treatment.

### DSS-induced colitis model

After the ligature had remained in place for 14 days, mice received 1.5% DSS (molecular mass ~40 000 kD; Sigma-Aldrich, UK) in their drinking water (tap water) for 14 days, after which they received normal tap water for a 2-day recovery period. The DAI was applied to measure the severity of colitis, which was scored as follows, as previously described: body weight loss (0, none; 1, 1%–5%; 2, 5%–10%; 3, 10%–20%; 4, >20%), stool consistency (2, loose stools; 4, diarrhea), and bleeding (2, positive haemoccult; 4, gross bleeding).^85^ The scores of individual measurements were added to calculate the DAI. Mice were sacrificed on day 28. Colons were collected, and their lengths were measured.

### Histological staining and histopathological evaluation

After collection, the maxillae and colons were fixed in 4% PFA. The colons were then paraffin-embedded and sectioned, followed by H&E staining. At the same time, the maxillae were decalcified in 0.5 mol·L^–1^ EDTA for 3 weeks, dehydrated in a 30% sucrose solution and embedded in Tissue-Tek optimum cutting temperature (OCT) compound (Sakura Finetek, Torrance, CA, USA). They were cut with a freezing microtome (Leica CM1900, Germany) and stained with H&E (Servicebio, Wuhan, China) as well as tartrate-resistant acid phosphatase (TRAP, 387A-1KT, Sigma-Aldrich). The distance between the cementoenamel junction and alveolar bone crest (CEJ-ABC distance) of the sections stained by H&E was measured to evaluate bone loss. TRAP-positive multinucleate cells were considered osteoclasts and a sign of bone resorption.

For assessment of colon inflammation, histological scores were determined by the following criteria: the severity of epithelial/crypt loss (score, 0-4) and the extent of inflammatory cell infiltration in the lamina propria (score, 0-4). Each score was multiplied by a factor representing the percentage of the colon involved (1, 0%–25%; 2, 26%–50%; 3, 51%–75%; 4, 76%–100%), and the scores on the individual measures were then added to calculate the overall histological score for each sample, as previously described.^41^

### Micro-CT

We collected the mouse maxillary bones from mice under each experimental condition and used them for three-dimensional high-resolution micro-CT analysis (Scano Micro-CT, μCT50, Switzerland). The key parameters were set as follows: 70 kV, 110 mA, and 7-μm increments. Three-dimensional microstructural image data were reconstructed and analyzed by using image analysis software (Mimics Research 21.0, Materialize, Belgium). The CEJ-ABC distance was measured at six sites, including mesial, middle, and distal points of both the buccal and palatal sides, and the mean CEJ-ABC distance was then calculated.

### T lymphocyte culture and differentiation

The mice were subjected to cervical dislocation following anesthesia with isoflurane to reduce their pain. They were then sterilized in 70% ethanol. The spleen was dissected by aseptic operation and then physically homogenized into pieces. The crushed spleen tissue was dissolved in a small amount of PBS solution and filtered with a 0.45-μm sieve. The filtered solution was added to 3 times the volume of RBC lysis buffer (CWBio), incubated on ice for 15 min, and vortexed slightly in the tube at 5-min intervals. After centrifugation at 450 × *g* for 5 min, the precipitated cells were suspended in RPMI-1640 medium (Gibco).

Naive CD4^+^ T cells were harvested from the spleen via the MojoSort™ Mouse CD4 Naive T-Cell Isolation Kit (BioLegend, San Diego, CA, USA) and seeded into 48-well plates (3 × 10^6^ per well) precoated with anti-CD3 (5 mg·mL^−1^) and anti-CD28 (2 mg·mL^−1^) solutions. To investigate the roles of miR-1246 in the expression of Nfat5 in CD4^+^ T cells, CD4^+^ T cells were incubated with miR-1246 mimics (RiboBio, Guangzhou, China) or inhibitors (RiboBio) for 24 h.

To examine the differentiation propensity, CD4^+^ T cells were cultured in Th17 differentiation media or Treg differentiation media. The Th17 differentiation medium contained 1.0 ng·mL^–1^ TGF-β, 30 ng·mL^–1^ IL-6, 20 ng·mL^–1^ IL-1β, 20 ng·mL^–1^ IL-23, 10 ng·mL^–1^ anti-IL-4 and 10 mg·mL^–1^ anti-IFN-γ. The Treg differentiation medium contained 50 ng·mL^–1^ IL-2 and 5 ng·mL^–1^ TGF-β. All cytokines were purchased from R&D Systems. T cells were further cultured with PBS, 2D-exos (10 μg of exosomes per 10^5^ cells), 3D-exos (10 μg of exosomes per 10^5^ cells), or 3D-exos with the miRNA inhibitor (RiboBio) in differentiation medium for 72 h and restimulated with PMA (Sigma-Aldrich) and ionomycin (Sigma-Aldrich) in the presence of brefeldin A (BD Biosciences, CA, USA) for 5 h before further analysis of intracellular cytokines. These cells were maintained in a standard 37 °C CO_2_ (5%) incubator. miRNA mimics and inhibitors were transfected with Lipofectamine 2000 (Invitrogen, CA, USA).

### RNA-seq

Total RNA was isolated from the gingivae of 3D-exo-treated, 2D-exo-treated, and PBS-treated mice with NucleoZOL reagent (Gene Company Limited, Hong Kong, China). RNA-seq libraries were prepared with the NEBNext® Ultra™ RNA Library Prep Kit (NEB, USA), followed by sequencing with Illumina Sequencing (HiSeq, Fasteris SA, Switzerland) at Novogene Co. Ltd. (Beijing, China). Small RNAs were isolated from the 3D-exos and 2D-exos for miRNA-seq. The miRNA-seq libraries were prepared and sequenced with an Illumina HiSeq platform at RiboBio Co. Ltd. (Guangzhou, China).

Feature counts were used to calculate read counts, and DESeq2 was used to analyse the differential expression of genes. Genes with a corrected *p*-value ≤ 0.05 and an absolute log2 (fold-change) > 2 were considered differentially expressed. GO enrichment analysis of the top 200 DEGs was performed with the Database for Annotation, Visualization and Integrated Discovery (DAVID).

### RNA extraction, reverse transcription, and RT-qPCR

Total RNA was extracted from the colons, the gingivae and CD4^+^ T cells with NucleoZOL reagent (Gene Company Limited) and was then reverse-transcribed into cDNA using PrimeScript RT Master Mix (TaKaRa, Ltd, Osaka, Japan). Real-time polymerase chain reaction (RT-qPCR) was performed to measure gene expression levels in a Bio-Rad CFX96™ detection system (Roche, Sweden) with Hieff qPCR SYBR Green Master Mix (Yeasen, Shanghai, China). Small RNA was isolated with a miRNA isolation kit (Qiagen, Hilden, Germany), and cDNA was prepared with a miRNA reverse transcription kit (Shenggong, Shanghai, China). RT-qPCR was performed to measure the expression level of genes in a Bio-Rad CFX96™ Detection System (Roche) with Hieff qPCR SYBR Green Master Mix (Yeasen). *U6* was applied as the internal reference. The primers used in the process are shown in Supplementary Table S1.

### Western blot analysis

After lysis in RIPA buffer (Millipore, Billerica, MA, USA) on ice for 30 min and centrifugation for 15 min, proteins were extracted from CD4^+^ T cells and tissues. A BCA protein assay kit (CWBio) was used to detect the total protein concentration. Sodium dodecyl sulfate-polyacrylamide gel electrophoresis (SDS-PAGE) was used to separate the proteins, which were then transferred onto poly (vinylidene fluoride) membranes (Millipore). The membranes were blocked in buffer containing 5% bovine serum albumin at room temperature for 30 min and then incubated with the primary antibodies at 4 °C overnight followed by horseradish peroxidase-conjugated secondary antibodies for 1 h at room temperature. The antibodies used in the process are shown in Supplementary Table S2.

### Tissue extraction and single-cell preparations

The gingival tissues were isolated and cut into small pieces, followed by enzymatic digestion with RPMI-1640 medium containing 4 mg/mL dispase and 3 mg/mL collagenase type I for 60 min at 37 °C. The digest solution was then filtered through a 70-μm cell strainer (Biologix Research Company, USA) to acquire a single-cell suspension.

### Flow cytometry

For surface antigen staining, single-cell suspensions were stained with the indicated antibodies at 4 °C for 30 min. Dead cells were removed using Zombie viability dye (BioLegend). For intracellular antigen staining, cells were fixed in fixation buffer (0.5 mL per tube; BioLegend) for 20 min after staining with surface antigen antibody. Then, they were stained with predetermined intracellular antibodies at 4 °C for 30 min. The gating strategies are shown in Figs. S3, S4, and S6. Data were acquired using CytoFlex (Beckman CytoFlex, USA) and analyzed with FlowJo V10.0 (TreeStar, Ashland, OR, USA).

### Luciferase activity assay

miR-1246-binding sites on the 3’-untranslated region (UTR) of *Nfat5* were recognized by TargetScan online bioinformatics software (http://www.targetscan.org). Dual luciferase activity assay was then measured to confirm the relationship between miR-1246 and Nfat5. In brief, Nfat5 recombinant plasmids (Nfat5-WT and Nfat5-Mut) and mimic NC or miR-1246 were transfected into 293T cells. The luciferase activity was ultimately analyzed using a dual luciferase assay kit (Promega Corporation, USA) following the manufacturer’s instructions.

### Statistics

All data are presented as the mean ± SEM of at least three independent experiments. After normality testing, all data were analyzed by 2-tailed unpaired Student’s *t*-test, the Kruskal–Wallis test (nonparametric samples) or 1-way ANOVA (parametric sample) followed by either Dunn’s test (nonparametric samples) or Tukey’s test (parametric samples) as the post hoc test. All statistical analyses were performed with GraphPad Prism software.

## Supplementary information

Supplementary data

## References

[1] Dubar M (2020). Relations of psychosocial factors and cortisol with periodontal and bacterial parameters: a prospective clinical study in 30 patients with periodontitis before and after non-surgical treatment. Int. J. Environ. Res. Public Health.

[2] Li H (2020). Low-intensity pulsed ultrasound upregulates osteogenesis under inflammatory conditions in periodontal ligament stem cells through unfolded protein response. Stem Cell Res. Ther..

[3] Byun SH, Lee S, Kang SH, Choi HG, Hong SJ (2020). Cross-sectional analysis of the association between periodontitis and cardiovascular disease using the Korean Genome and Epidemiology Study Data. Int. J. Environ. Res. Public Health.

[4] Liccardo D (2019). Periodontal disease: a risk factor for diabetes and cardiovascular disease. Int. J. Mol. Sci..

[5] Dominy SS (2019). Porphyromonas gingivalis in Alzheimer’s disease brains: evidence for disease causation and treatment with small-molecule inhibitors. Sci. Adv..

[6] Lin J (2019). Influence of adjacent teeth absence or extraction on the outcome of non-surgical periodontal therapy. Int. J. Environ. Res. Public Health.

[7] Ho W, Eubank T, Leblebicioglu B, Marsh C, Walters J (2010). Azithromycin decreases crevicular fluid volume and mediator content. J. Dent. Res..

[8] Kornsuthisopon C, Pirarat N, Osathanon T, Kalpravidh C (2020). Autologous platelet-rich fibrin stimulates canine periodontal regeneration. Sci. Rep..

[9] Hajishengallis G (2014). Immunomicrobial pathogenesis of periodontitis: keystones, pathobionts, and host response. Trends Immunol..

[10] Loos BG, Van Dyke TE (2020). The role of inflammation and genetics in periodontal disease. Periodontol.

[11] Chew JRJ (2019). Mesenchymal stem cell exosomes enhance periodontal ligament cell functions and promote periodontal regeneration. Acta Biomater..

[12] Zhang Q (2018). Exosomes originating from MSCs stimulated with TGF-β and IFN-γ promote Treg differentiation. J. Cell. Physiol..

[13] Chang W, Wang J (2019). Exosomes and their noncoding rna cargo are emerging as new modulators for diabetes mellitus. Cells.

[14] Wu R (2019). Exosomes secreted by urine-derived stem cells improve stress urinary incontinence by promoting repair of pubococcygeus muscle injury in rats. Stem Cell Res. Ther..

[15] Ariston Gabriel AN (2020). The involvement of exosomes in the diagnosis and treatment of pancreatic cancer. Mol. Cancer.

[16] Liu L (2021). Bone marrow mesenchymal stem cell-derived small extracellular vesicles promote periodontal regeneration. Tissue Eng. Part A.

[17] Novello S, Pellen‐Mussi P, Jeanne S (2021). Mesenchymal stem cell‐derived small extracellular vesicles as cell‐free therapy: Perspectives in periodontal regeneration. J. Periodontal Res..

[18] Shen WC (2019). Methylation and PTEN activation in dental pulp mesenchymal stem cells promotes osteogenesis and reduces oncogenesis. Nat. Commun..

[19] Alge DL (2009). Donor-matched comparison of dental pulp stem cells and bone marrow-derived mesenchymal stem cells in a rat model. J. Tissue Eng. Regen. Med.

[20] Aurrekoetxea M (2015). Dental pulp stem cells as a multifaceted tool for bioengineering and the regeneration of craniomaxillofacial tissues. Front. Physiol..

[21] Gao X (2020). Effects of targeted delivery of metformin and dental pulp stem cells on osteogenesis via demineralized dentin matrix under high glucose conditions. ACS Biomater. Sci. Eng..

[22] Xie Z (2021). Functional dental pulp regeneration: basic research and clinical translation. Int. J. Mol. Sci..

[23] Li J (2020). A decellularized matrix hydrogel derived from human dental pulp promotes dental pulp stem cell proliferation, migration, and induced multidirectional differentiation in vitro. J. Endod..

[24] Tu S (2020). LncRNA CALB2 sponges miR-30b-3p to promote odontoblast differentiation of human dental pulp stem cells via up-regulating RUNX2. Cell. Signal..

[25] Shen Z (2020). Chitosan hydrogel incorporated with dental pulp stem cell-derived exosomes alleviates periodontitis in mice via a macrophage-dependent mechanism. Bioact. Mater..

[26] Zhang Y (2020). Exosome: a review of its classification, isolation techniques, storage, diagnostic and targeted therapy applications. Int. J. Nanomed..

[27] Phan J (2018). Engineering mesenchymal stem cells to improve their exosome efficacy and yield for cell-free therapy. J.Extracell. Vesicles.

[28] Haraszti RA (2018). Exosomes produced from 3D cultures of MSCs by tangential flow filtration show higher yield and improved activity. Mol. Ther..

[29] Yan L, Wu X (2020). Exosomes produced from 3D cultures of umbilical cord mesenchymal stem cells in a hollow-fiber bioreactor show improved osteochondral regeneration activity. Cell Biol. Toxicol..

[30] Cao J (2020). Three-dimensional culture of MSCs produces exosomes with improved yield and enhanced therapeutic efficacy for cisplatin-induced acute kidney injury. Stem Cell Res. Ther..

[31] Yang L, Zhai Y, Hao Y, Zhu Z, Cheng G (2020). The regulatory functionality of exosomes derived from hUMSCs in 3D culture for Alzheimer’s disease therapy. Small.

[32] Papageorgiou SN (2017). Inflammatory bowel disease and oral health: systematic review and a meta-analysis. J. Clin. Periodontol..

[33] Baima G (2021). Shared microbiological and immunological patterns in periodontitis and IBD: a scoping review. Oral Dis..

[34] Koliarakis I (2019). Oral bacteria and intestinal dysbiosis in colorectal cancer. Int. J. Mol. Sci..

[35] Lira-Junior R, Figueredo CM (2016). Periodontal and inflammatory bowel diseases: Is there evidence of complex pathogenic interactions?. World J. Gastroenterol..

[36] Vavricka SR (2013). Periodontitis and gingivitis in inflammatory bowel disease. Inflamm. Bowel Dis..

[37] Yu H-C, Chen T-P, Chang Y-C (2018). Inflammatory bowel disease as a risk factor for periodontitis under Taiwanese National Health Insurance Research database. J. Dent. Sci..

[38] She Y (2020). Periodontitis and inflammatory bowel disease: a meta-analysis. BMC Oral. Health.

[39] Kang EA (2020). Periodontitis combined with smoking increases risk of the ulcerative colitis: a national cohort study. World J. Gastroenterol..

[40] Meghil MM, Cutler CW (2020). Oral microbes and mucosal dendritic cells, “spark and flame” of local and distant inflammatory diseases. Int. J. Mol. Sci..

[41] Kitamoto S (2020). The intermucosal connection between the mouth and gut in commensal pathobiont-driven colitis. Cell.

[42] Koh K (2020). UBA2 activates Wnt/β-catenin signaling pathway during protection of R28 retinal precursor cells from hypoxia by extracellular vesicles derived from placental mesenchymal stem cells. Stem Cell Res. Ther..

[43] Pisano S (2020). Immune (Cell) Derived exosome mimetics (IDEM) as a treatment for ovarian cancer. Front. Cell Dev. Biol..

[44] Muntasell A, Berger AC, Roche PA (2007). T cell-induced secretion of MHC class II–peptide complexes on B cell exosomes. EMBO J..

[45] Zhu X (2019). Macrophages derived exosomes deliver miR-223 to epithelial ovarian cancer cells to elicit a chemoresistant phenotype. J. Exp. Clin. Cancer Res..

[46] Suh JS (2020). Rosuvastatin prevents the exacerbation of atherosclerosis in ligature-induced periodontal disease mouse model. Sci. Rep..

[47] Offenbacher S (2018). GWAS for interleukin-1β levels in gingival crevicular fluid identifies IL37 variants in periodontal inflammation. Nat. Commun..

[48] Takahashi N (2016). Neuronal TRPV1 activation regulates alveolar bone resorption by suppressing osteoclastogenesis via CGRP. Sci. Rep..

[49] Yan Y (2018). Artemisinin analogue SM934 ameliorates DSS-induced mouse ulcerative colitis via suppressing neutrophils and macrophages. Acta Pharmacol. Sin..

[50] Rangan P (2019). Fasting-mimicking diet modulates microbiota and promotes intestinal regeneration to reduce inflammatory bowel disease pathology. Cell Rep..

[51] Sinha SR (2020). Dysbiosis-induced secondary bile acid deficiency promotes intestinal inflammation. Cell Host Microbe.

[52] Elashiry M (2020). Dendritic cell derived exosomes loaded with immunoregulatory cargo reprogram local immune responses and inhibit degenerative bone disease in vivo. J. Extracell. Vesicles.

[53] Gaffen SL, Moutsopoulos NM (2020). Regulation of host-microbe interactions at oral mucosal barriers by type 17 immunity. Sci. Immunol..

[54] Roda G (2020). Crohn’s disease. Nat. Rev. Dis. Prim.

[55] Mousset CM (2019). Comprehensive phenotyping of T cells using flow cytometry. Cytom. Part A.

[56] Tian J (2020). Olfactory ecto-mesenchymal stem cell-derived exosomes ameliorate experimental colitis via modulating Th1/Th17 and Treg cell responses. Front. Immunol..

[57] Zheng Y (2019). Exosomal microRNA-155-5p from PDLSCs regulated Th17/Treg balance by targeting sirtuin-1 in chronic periodontitis. J. Cell. Physiol..

[58] Bayraktar R, Van Roosbroeck K, Calin GA (2017). Cell‐to‐cell communication: microRNAs as hormones. Mol. Oncol..

[59] Deng G (2019). Foxp3 post-translational modifications and Treg suppressive activity. Front. Immunol..

[60] Lee J-Y (2020). Serum amyloid A proteins induce pathogenic Th17 cells and promote inflammatory disease. Cell.

[61] Lee N, Kim D, Kim WU (2019). Role of NFAT5 in the immune sustem and pathogenisis of autoimmune disease. Front. Immunol..

[62] Zhao J (2019). Mesenchymal stromal cell-derived exosomes attenuate myocardial ischaemia-reperfusion injury through miR-182-regulated macrophage polarization. Cardiovasc. Res..

[63] Shi C (2020). Regulating the balance of Th17/Treg cells in gut-lung axis contributed to the therapeutic effect of Houttuynia cordata polysaccharides on H1N1-induced acute lung injury. Int. J. Biol. Macromol..

[64] Rajendran M (2019). Systemic antibiotic therapy reduces circulating inflammatory dendritic cells and Treg–Th17 plasticity in periodontitis. J. Immunol..

[65] Ha DH (2020). Mesenchymal stem/stromal cell-derived exosomes for immunomodulatory therapeutics and skin regeneration. Cells.

[66] Kim HY (2020). Mesenchymal stem cell-derived magnetic extracellular nanovesicles for targeting and treatment of ischemic stroke. Biomaterials.

[67] Jauković A (2020). Specificity of 3D MSC spheroids microenvironment: impact on MSC behavior and properties. Stem Cell Rev. Rep..

[68] Guo S (2021). Stimulating extracellular vesicles production from engineered tissues by mechanical forces. Nano Lett..

[69] Bakheet SA (2019). CXCR3 antagonist AMG487 suppresses rheumatoid arthritis pathogenesis and progression by shifting the Th17/Treg cell balance. Cell. Signal..

[70] Yan J, Luo M, Chen Z, He B (2020). The function and role of the Th17/Treg cell balance in inflammatory bowel disease. J. Immunol. Res..

[71] Karthikeyan B, Talwar, Arun KV, Kalaivani S (2015). Evaluation of transcription factor that regulates T helper 17 and regulatory T cells function in periodontal health and disease. J. Pharm. Bioallied Sci..

[72] Windsor JW, Kaplan GG (2019). Evolving epidemiology of IBD. Curr. Gastroenterol. Rep..

[73] Neurath MF (2017). Current and emerging therapeutic targets for IBD. Nat. Rev. Gastroenterol. Hepatol..

[74] Dickey LL, Worne CL, Glover JL, Lane TE, O’Connell RM (2016). MicroRNA-155 enhances T cell trafficking and antiviral effector function in a model of coronavirus-induced neurologic disease. J. Neuroinflammation.

[75] Holmstrøm K, Pedersen AE, Gad M (2017). Analysis of miR-146a and miR-142-3p as potential markers of freshly isolated or in vitro -expanded human Treg cells. Scand. J. Immunol..

[76] Aramburu J, López-Rodríguez C (2019). Regulation of inflammatory functions of macrophages and T lymphocytes by NFAT5. Front. Immunol..

[77] Lee JU, Kim LK, Choi JM (2018). Revisiting the concept of targeting NFAT to control T cell immunity and autoimmune diseases. Front. Immunol..

[78] Qiu, W. et al. Exosomal miR-1246 from glioma patient body fluids drives the differentiation and activation of myeloid-derived suppressor cells. *Mol. Ther*. S1525-0016(21)00353-1 (2021). 10.1016/j.ymthe.2021.06.023 PMC8636176 34217892

[79] Cesarz Z, Tamama K (2016). Spheroid culture of mesenchymal stem cells. Stem Cells Int.

[80] ZHANG Q (2015). p53-induced microRNA-1246 inhibits the cell growth of human hepatocellular carcinoma cells by targeting NFIB. Oncol. Rep..

[81] Oikawa T (2018). Necessity of p53-binding to the CDH1 locus for its expression defines two epithelial cell types differing in their integrity. Sci. Rep..

[82] Pomo JM, Taylor RM, Gullapalli RR (2016). Influence of TP53 and CDH1 genes in hepatocellular cancer spheroid formation and culture: a model system to understand cancer cell growth mechanics. Cancer Cell Int..

[83] Imhann F (2016). Proton pump inhibitors affect the gut microbiome. Gut.

[84] Atarashi K (2017). Ectopic colonization of oral bacteria in the intestine drives T H 1 cell induction and inflammation. Science (80-.).

[85] Zhou C (2020). Immunomodulatory effect of urine-derived stem cells on inflammatory bowel diseases via downregulating th1/th17 immune responses in a PGE2-dependent manner. J. Crohn’s Colitis.

