# Supplementary material for: Exosomes derived from 3D-cultured MSCs improve therapeutic effects in periodontitis and experimental colitis and restore the Th17 cell/Treg balance in inflamed periodontium
Source: Int J Oral Sci. 2021 Dec 14;13:43. doi: 10.1038/s41368-021-00150-4 (PMC8671433; doi:10.1038/s41368-021-00150-4)
Supplement: Supplementary file 1 — Supplementary data [file 41368_2021_150_MOESM1_ESM.pdf]

Supplementary data

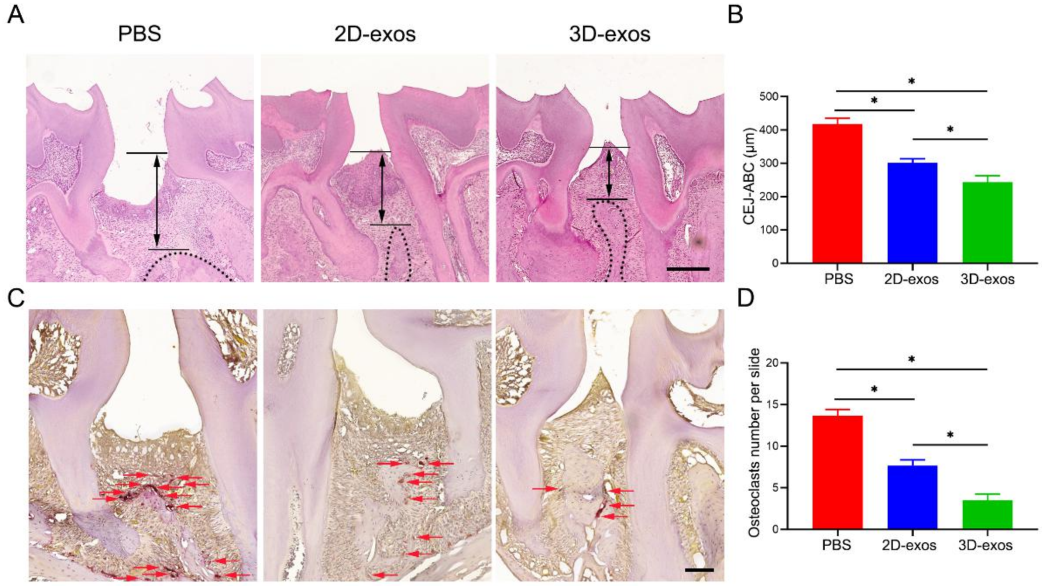

Figure S1. 3D-exos exerted enhanced effects in ameliorating periodontitis

(A) Histological sections of the periodontium from the PBS-, 2D-exo- and 3D-exo-treated groups stained with H&E are shown. The vertical line extends from the CEJ to the ABC. Scale bar = 250 μm. (B) Statistical analysis of the CEJ-ABC distance in each group (n = 6), which was quantified in each microscope field of view. The error bars represent the SEM. *$p < 0.05$. (C) Histological sections of the periodontium from the PBS-, 2D-exo- and 3D-exo-treated groups stained with TRAP are shown. Osteoclasts are stained red and marked by red arrows. Scale bar = 50 μm. (D) Statistical analysis of the number of osteoclasts in each microscope field of view in each group (n = 6), as determined by TRAP staining. The error bars represent the SEM. *$p < 0.05$.

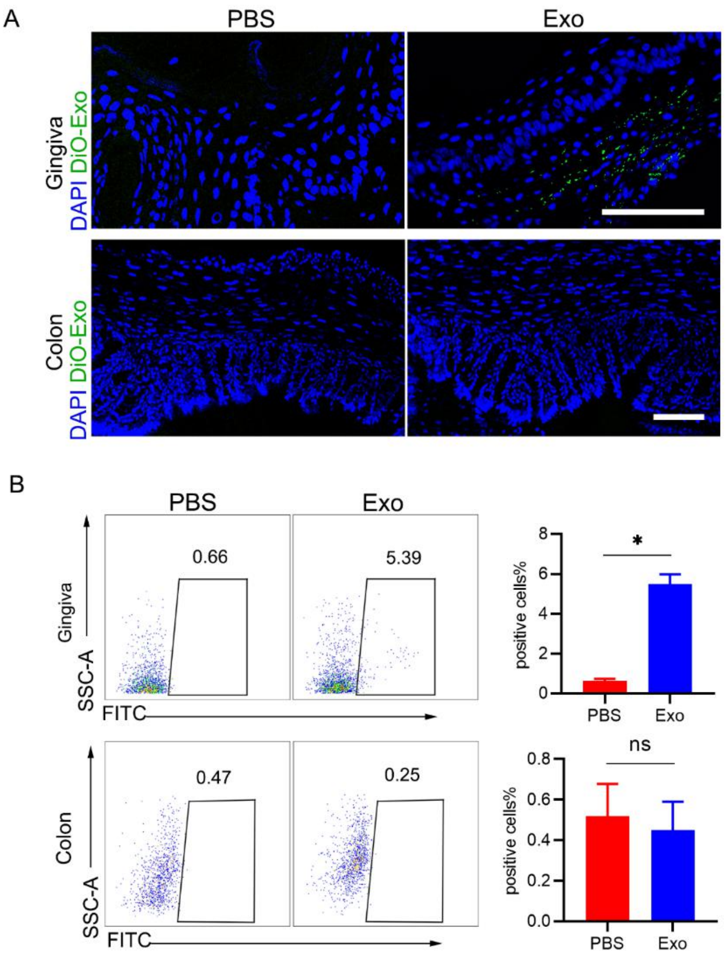

Figure S2. Locally administrated exosomes were detected in the maxillary gingiva but were barely detectable in colon

(A) Representative fluorescence images of gingiva and colon. Nuclei were stained with DAPI. Scale bar = 100 μm. (B) Flow cytometry analysis for the percentage of DiO-Exo-positive cells in gingiva and colon. The error bars represent the SEM. $*p < 0.05$.

A

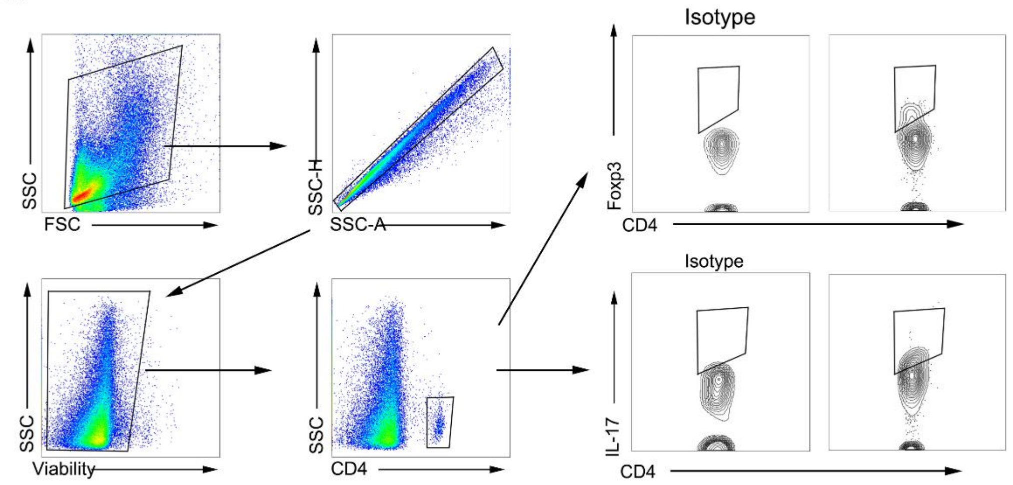

Figure S3. Gating strategies for the flow cytometry analysis of gingival cells

(A) Gating strategy used to select single, live $CD4^{+}$ $Foxp3^{+}$ cells and single, live $CD4^{+}$ $IL\text{-}17^{+}$ cells in the $CD4^{+}$ cell population and the isotype control.

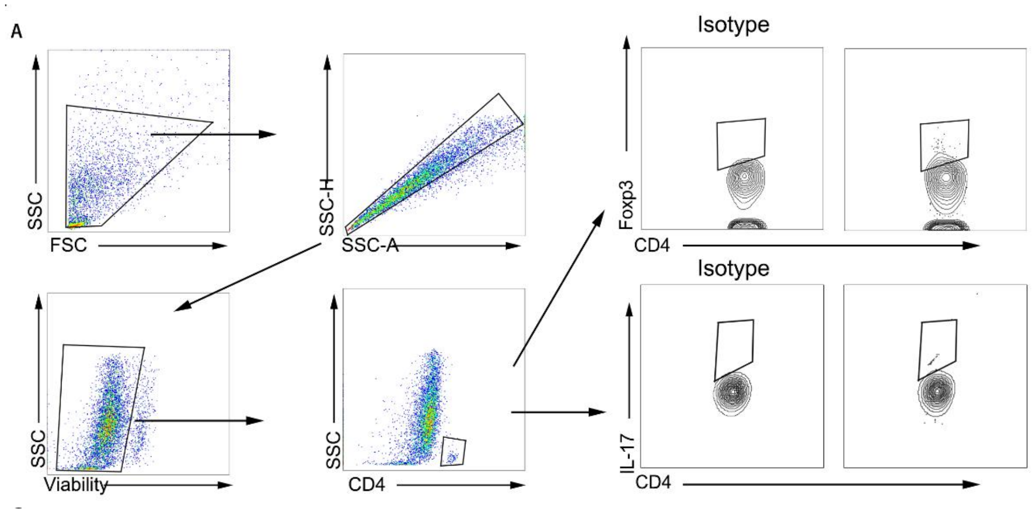

Figure S4. Gating strategies for the flow cytometry analysis of colon cells

(A) Gating strategy used to select single, live $CD4^+$ $Foxp3^+$ cells and single, live $CD4^+$ $IL\text{-}17^+$ cells in the $CD4^+$ cell population and the isotype control.

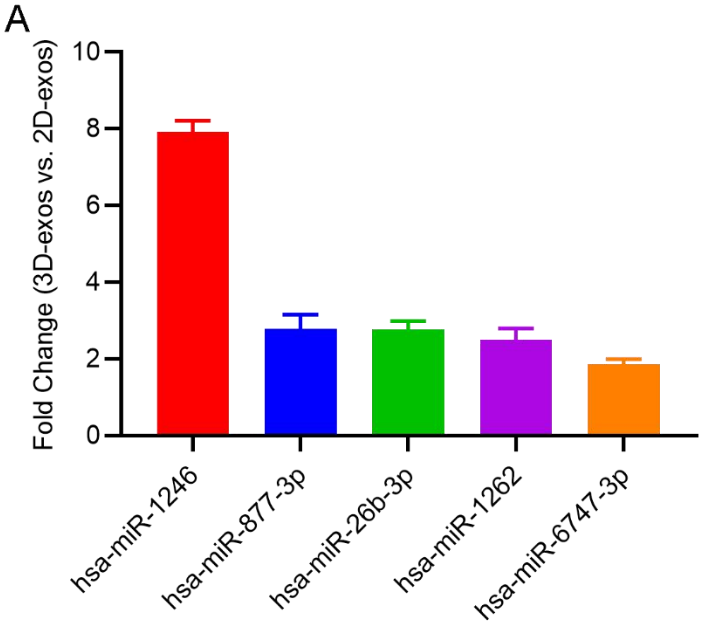

Figure S5. miR-1246 levels in 3D-exos and 2D-exos

(A) Fold changes in miR-1246 levels in 3D-exos and 2D-exos were analysed by RT-qPCR (n = 6 per group). The error bars represent the SEM.

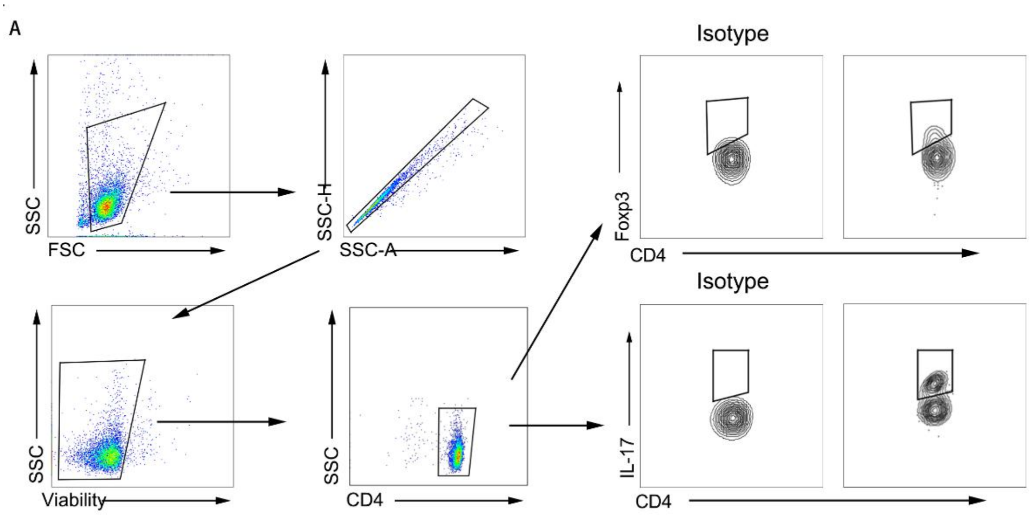

Figure S6. Gating strategies for the flow cytometry analysis of CD4⁺ T cells *in vitro*

(A) Naïve CD4⁺ T cells were isolated from splenocytes, stimulated with the indicated cytokines to induce Treg and Th17 cell formation *in vitro* and further cultured with PBS, NCI-3D-exos or miR1246I-3D-exos. Gating strategy used to select single, live CD4⁺ Foxp3⁺ cells and single, live CD4⁺ IL-17⁺ cells in the CD4⁺ cell population and the isotype control.

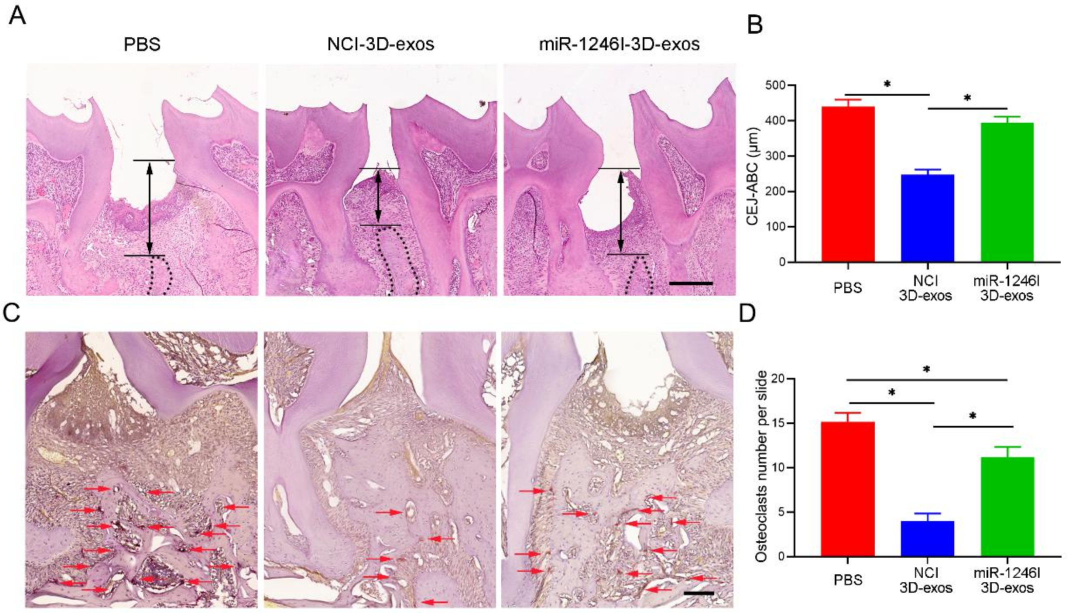

Figure S7. Antagomir-1246 reversed the effects of DPSC 3D-exos in mitigating periodontitis

(A) Histological sections of the periodontium from the PBS-, NCI-3D-exo- and miR1246I-3D-exo-treated groups stained with H&E are shown. The vertical line extends from the CEJ to the ABC. Scale bar = 250 μm. (B) Statistical analysis of the CEJ-ABC distance in each group (n = 6), which was quantified in each microscope field of view. Error bars represent the SEM. *$p < 0.05$. (C) Histological sections of the periodontium from the PBS-, NCI-3D-exo- and miR1246I-3D-exo-treated groups stained with TRAP are shown. Osteoclasts are stained red and marked by red arrows. Scale bar = 50 μm. (D) Statistical analysis of the number of osteoclasts in each group (n = 6) in each microscope field of view, as determined by TRAP staining. The error bars represent the SEM. *$p < 0.05$.

Table S1. Primers used in this study

GENE	PRIMER SEQUENCE (5'-3')
TNF-α	CCCTCACACTCAGATCATCTTCT
	GCTACGACGTGGGCTACAG
IL -1β	GCAACTGTTCCTGAACTCAACT
	ATCTTTTGGGGTCCGTCAACT
IL-6	TAGTCCTTCCTACCCCAATTTCC
	TTGGTCCTTAGCCACTCCTTC
GAPDH	AGGTCGGTGTGAACGGATTTG
	TGTAGACCATGTAGTTGAGGTCA
	CAGCGCCCAATAGTTGGCA
	TGCTGGTGAAAAATTGACTGGT
Foxp3	CCCATCCCCAGGAGTCTTG
	ACCATGACTAGGGGCACTGTA
ROR-γt	GACCCACACCTCACAAATTGA
	AGTAGGCCACATTACACTGCT
U6	GCGCGTCGTGAAGCGTTC
hsa-miR-1246	AATGGATTTTTGGAGCAGG
hsa-miR-877-3p	GACCCUCCUCCCUCUUCUCCU
hsa-miR-26b-3p	CUCGGUUCAUUACCUCUUGUCC
hsa-miR-1262	UAGGAAGAUGUUUAAGUGGGUA
hsa-miR-6747-3p	GACCACGUCUCCUUCCGUCCU

Table S2. Antibodies used in this study

MARKER (SPECIES)	DILUTION	DISTRIBUTOR/SOURCE (CATALOGUE NUMBER)
Primary antibodies:		
CD9 Rabbit pAb	1:1000(WB)	Affinity (DF6565)
CD63 Rabbit mAb	1:1000 (WB)	Affinity (DF2305)
GM130 Rabbit mAb	1:1000 (WB)	Affinity (DF7286)
TSG101 Rabbit mAb	1:1000 (WB)	Affinity (DF8427)
NFAT5 Rabbit mAb	1:1000 (WB)	Zen-Bio (251746)
β-Tubulin Mouse mAb (4F2)	1:5000	EMAR (EM31013)
Secondary antibodies:		
Anti-mouse IgG HRP-linked Ab	1:5000	CST (7076)
Anti-rabbit IgG HRP-linked Ab	1:5000	CST (7074)
Anti-rabbit IgG (Fluor® 488 Conjugate)	1:500	CST (4412)
Anti-mouse IgG (Fluor® 488 Conjugate)	1:500	CST (4408)
Antibodies for flow cytometry:		
CD63-APC Ab	1:100	BioLegend (143905)
CD9-APC Ab	1:100	BioLegend (124811)
CD4 -PE Ab	1:100	BioLegend (100407)
IL-17 -AF488 Ab	1:100	BioLegend (516603)
FOXP3-AF647 Ab	1:100	BioLegend (320113)
Isotype Ctrl-PE Ab	1:100	BioLegend (400607)
Isotype Ctrl- PE/CY7 Ab	1:100	BioLegend (400617)
Isotype Ctrl- AF488 Ab	1:100	BioLegend (400417)
Isotype Ctrl- APC Ab	1:100	BioLegend (400713)

Table S3. The experimental groups information in this study

GROUP CODE (6 mice for each group)	TREATMENT
PBS-treated DSS-P mice (PBS-treated group)	After 14 days of periodontal ligature were treated with 1.5% DSS and PBS for another 14 days
2D-exo-treated DSS-P mice (2D-exo-treated group)	After 14 days of periodontal ligature were treated with 1.5% DSS and 2D-exos (50 µg/mouse) for another 14 days
3D-exo-treated DSS-P mice (3D-exo-treated group)	After 14 days of periodontal ligature were treated with 1.5% DSS and 3D-exos (50 µg/mouse) for another 14 days
NCI-3D-exos-treated DSS-P mice (NCI-3D-exo group)	After 14 days of periodontal ligature were treated with 1.5% DSS and 3D-exos with NC antagomir (50 µg/mouse) for another 14 days
miR1246I-3D-exos-treated DSS-P mice (miR1246I-3D-exo group)	After 14 days of periodontal ligature were treated with 1.5% DSS and 3D-exos with miR-1246 antagomir (50 µg/mouse) for another 14 days
